# Optimization of Quantitative PCR Methods for Enteropathogen Detection

**DOI:** 10.1371/journal.pone.0158199

**Published:** 2016-06-23

**Authors:** Jie Liu, Jean Gratz, Caroline Amour, Rosemary Nshama, Thomas Walongo, Athanasia Maro, Esto Mduma, James Platts-Mills, Nadia Boisen, James Nataro, Doris M. Haverstick, Furqan Kabir, Paphavee Lertsethtakarn, Sasikorn Silapong, Pimmada Jeamwattanalert, Ladaporn Bodhidatta, Carl Mason, Sharmin Begum, Rashidul Haque, Ira Praharaj, Gagandeep Kang, Eric R. Houpt

**Affiliations:** 1 Division of Infectious Diseases and International Health, University of Virginia, Charlottesville, Virginia, United States of America; 2 Haydom Global Health Institute, Haydom, Tanzania; 3 Kilimanjaro Clinical Research Institute, Moshi, Tanzania; 4 Department of Pediatrics, University of Virginia, Charlottesville, Virginia, United States of America; 5 Department of Pathology, University of Virginia, Charlottesville, Virginia, United States of America; 6 Department of Pediatrics and Child Health, Aga Khan University, Karachi, Pakistan; 7 Department of Enteric Diseases, Armed Forces Research Institute of Medical Sciences (AFRIMS), Bangkok, Thailand; 8 International Centre for Diarrhoeal Disease Research, Bangladesh (ICDDR,B), Dhaka, Bangladesh; 9 Christian Medical College, Vellore, Tamil Nadu, India; Hong Kong Institute for the Humanities and Social Sciences, HONG KONG

## Abstract

Detection and quantification of enteropathogens in stool specimens is useful for diagnosing the cause of diarrhea but is technically challenging. Here we evaluate several important determinants of quantification: specimen collection, nucleic acid extraction, and extraction and amplification efficiency. First, we evaluate the molecular detection and quantification of pathogens in rectal swabs versus stool, using paired flocked rectal swabs and whole stool collected from 129 children hospitalized with diarrhea in Tanzania. Swabs generally yielded a higher quantification cycle (Cq) (average 29.7, standard deviation 3.5 vs. 25.3 ± 2.9 from stool, *P*<0.001) but were still able to detect 80% of pathogens with a Cq < 30 in stool. Second, a simplified total nucleic acid (TNA) extraction procedure was compared to separate DNA and RNA extractions and showed 92% (318/344) sensitivity and 98% (951/968) specificity, with no difference in Cq value for the positive results (ΔCq_(DNA+RNA-TNA)_ = -0.01 ± 1.17, *P* = 0.972, N = 318). Third, we devised a quantification scheme that adjusts pathogen quantity to the specimen’s extraction and amplification efficiency, and show that this better estimates the quantity of spiked specimens than the raw target Cq. In sum, these methods for enteropathogen quantification, stool sample collection, and nucleic acid extraction will be useful for laboratories studying enteric disease.

## Introduction

Detection of enteropathogens in stool samples is important for the diagnosis of diarrhea and other enteric infections but is technically challenging. Firstly, stool samples may not always be available for collection, thus obtaining rectal swabs is an appealing alternative for its ready availability. Recently, swabs have been shown to allow detection of some enteropathogens by PCR [1, 2] but have not been compared to stool for a wide panel of pathogens. Additionally, since enteropathogens cover a broad range of microorganisms, including both DNA and RNA viruses, bacteria, fungi, protozoa, and helminths, separate DNA and RNA extraction protocols are often used, with RNA extraction usually performed on a stool supernatant. For highly multiplexed molecular panels, it would be favorable to use a single extraction method that can efficiently isolate total nucleic acid from the entire sample. Finally, asymptomatic carriage of enteropathogens is common in resource-limited settings, particularly when sensitive molecular assays are used [3]. We have found that quantification is useful for inferring diarrhea etiology [4, 5]. However, the qPCR quantification cycle is assay and platform dependent and is affected by the presence of stool inhibitors, making it difficult to directly compare results across studies where different technologies have been used. In this work therefore, we evaluate the utility of rectal swabs, develop a total nucleic acid extraction method, and demonstrate a quantification scheme for enteropathogens.

## Materials and Methods

### Sample collection

For evaluation of assay performance, genomic materials or reference strains were obtained from American Tissue and Culture Collection (ATCC, Manassas, VA) or BEI resources for adenovirus 1, 5, 40 and 41, human cytomegalovirus, enterovirus 71, Epstein-Barr virus, *Aeromonas hydrophila*, *Bacteroides fragilis*, *Campylobacter coli*, *Campylobacter upsalensis*, *Campylobacter hyointestinalis*, *Campylobacter jejuni*, *Helicobacter pylori*, *Listeria monocytogenes*, *Mycobacterium tuberculosis*, *Plesiomonas shigelloides*, *Salmonella enterica*, *Vibrio parahaemolyticus*, *Yersinia enterocolitica*, *Blastocystis hominis*, *Cryptosporidium hominis*, *Cryptosporidium meleagridis*, *Schistosoma mansoni*. *Cryptosporidium parvum* and *Encephalitozoon intestinalis* were purchased from Waterborne Inc. (New Orleans, LA). PCR amplicons were generated from the relevant positive clinical samples for *Ancyclostoma duodenale*, *Necator americanus*, *Strongyloides stercoralis*, *Cyclospora cayetanensis*, *Cystoisospora belli*, and *Enterocytozoon bieneusi*. For comparison between stool and swab (FLOQSwabs; Copan Italia, Brescia, Italy), 129 consecutive swab samples were collected from children under five admitted for acute diarrhea in Haydom Lutheran Hospital, Tanzania. A matched stool sample from the same patient was obtained as soon as feasible within the same day. Raw stool samples were transported with a cold chain to the lab within 6 hours and stored at -80°C until testing. Swabs were stored at room temperature until testing. For comparison between different extraction methods and validation of the newly developed qPCR assays on clinical samples, we chose 246 archived stool samples collected in Tanzania, Bangladesh, Nepal, Pakistan, and India through the MAL-ED project (the Etiology, Risk Factors, and Interactions of Enteric Infections and Malnutrition and the Consequences for Child Health and Development [6]) in order to obtain specimens positive for 30 diverse enteropathogens. All sites including Haydom Global Health Institute, Tanzania, Aga Khan University, Pakistan, Armed Forces Research Institute of Medical Sciences, Thailand, International Centre for Diarrhoeal Disease Research, Bangladesh, Christian Medical College, India, received ethical approval from their respective governmental, local institutional, and collaborating institutional ethics review boards. Written informed consent was obtained from the parent or guardian of every child.

### Nucleic acid extraction

Spiked stool samples, clinical stool samples, and swabs were extracted with the QIAamp Stool DNA Mini kit (Qiagen, Valencia, CA), after a bead beating step and 95°C incubation. For stool specimens, 200 mg of raw stool was first lysed with QIAamp ASL buffer, beaten for 2 min with 212 to 300-μm glass beads (Sigma, St. Louis, MO), and incubated at 95°C for 5 min. The samples were centrifuged at full speed for 1 min to pellet stool particles, then 400 μl of ASL lysate were extracted and eluted in 200 μl of elution buffer following the manufacturer’s instructions. For swabs, the dry swab was mixed with the lysis buffer and glass beads, then subjected to bead beating directly and extracted following the same procedure as that for stool. Two extrinsic controls, Phocine Herpesvirus (PhHV) and bacteriophage MS2, were spiked into lysis buffer to monitor extraction and amplification efficiency. For comparison of extraction methods, one aliquot of each sample was extracted with QIAamp Stool DNA Mini kit and another aliquot was extracted with the QIAamp Viral RNA mini kit (Qiagen) or QuickGene RNA Tissue kit (FujiFilm, Tokyo, Japan). During all extractions an extraction blank was incorporated to monitor for lab contamination.

### PCR testing

20 μl of DNA extract, or 40 μl of 1:1 (in volume) DNA:RNA extract, was mixed with 50 μl of AgPath-ID One-Step RT-PCR buffer, 4 μl of enzyme mix, and nuclease free water to a 100-μl final volume. The reaction mixture underwent quantitative PCR using a TaqMan Array Card (TAC) platform as described previously [7]. Briefly, TAC is a microfluidic card compartmentalizing 384 individual TaqMan probe based real time PCR reactions. Eight samples were tested with 48 assays on each card. Quantification cycle (Cq) cutoffs of 35 were applied throughout as described [4]. Positive results were considered valid only when the corresponding extraction blank was negative for the relevant target; negative results were considered valid only when the extrinsic controls were positive for the given samples. Gene targets and PCR assays were adapted from publications whenever possible, with modifications if needed to the primers and probes to optimize their performance under the universal TAC cycling condition (S1 Table). Assays were validated on plates with the TaqMan array universal formula of a final primer concentration of 900 nM and a probe concentration of 250 nM. Linearity, intra-assay precision, inter-assay precision, limit of detection, and specificity were evaluated as outlined previously [4, 7].

### Combined positive controls and generation of standard curves

Six combined positive control plasmids, four for DNA targets and two for RNA targets, were designed as described [7, 8] and synthesized by GeneWiz (South Plainfield, NJ). For DNA targets the plasmids were directly utilized, while RNA templates were generated by PCR amplifying the insert portion with M13 primers and in vitro transcribing with T7 polymerase (Epicentre, Madison, WI). These plasmids and in vitro transcripts were pooled to a final concentration of 5×10^6^ copies per microliter, from which 10-fold serial dilutions were prepared and run on TaqMan Array cards in triplicate.

### Calculation of target copy numbers

In vitro transcripts for viruses, genomic DNA for bacteria and protozoa, and PCR amplicons for helminths, all of known copy number, were spiked into lysis buffer in different stool lots to mimic diverse inhibition conditions. Extracts were assayed on TAC. Standard curves of all the targets including extrinsic controls were generated by testing the combined positive controls in triplicate. Copy numbers of the spiked samples for a given target were then calculated based on the standard curves and adjusted to the efficiency derived from MS2 for RNA targets and PhHV for DNA targets (i.e. MS2 or PhHV copy number in the sample divided by the starting MS2 or PhHV copy numbers).

### Statistics

Pathogen quantity Cqs between swab and stool and between total nucleic acid extraction and combined DNA and RNA extracts were compared with Wilcoxon Signed Rank Test. Pathogen quantity Cqs between samples were compared with Mann-Whitney U test. Linear correlation between Cqs was measured by Pearson coefficient. Two-tailed p values were calculated, and values of <0.05 were considered statistically significant. All analyses were performed using SPSS version 23.

## Results

### Rectal swab versus stool samples

Swabs were first generated by spiking them into stool suspensions containing viral (rotavirus), bacterial (*Salmonella enterica*), and parasitic (*Cryptosporidium parvum* oocyst) targets. They were stored at -80°C, 4°C, and room temperature for 21 days dry or in eNAT medium (Copan Italia, Brescia, Italy), respectively (N = 3 for each), and no significant difference in yield was observed for the three targets (within 1.5 Cq). Therefore for the clinical study of paired swab and stool collection from129 consecutive patients in Haydom, Tanzania, swabs were stored dry at room temperature for an average of 17.6 ± 18.0 days before testing. Nucleic acid was extracted (details below) and tested by TaqMan array card for 33 pathogens. Extrinsic controls were positive in 93.8% and 93.0% of stool and swab samples, respectively (P = NS), with average Cq of 29.0 ± 2.0 versus 29.3 ± 2.1 (P = 0.090). At least one pathogen was identified in 128 of the stool and 123 of the swab samples (average 4.7±1.9 vs. 3.4±1.9 pathogens per sample, *P* < 0.001), respectively. Taking all 643 positive results for enteropathogens from the 129 stools and 129 swabs (Table 1), 373 were concordant between two sample types, 215 were detections in stool only, and 55 were detections in swabs only. Overall, swab samples yielded higher Cqs for most targets versus the corresponding stool (average ΔCq = 3.9 ± 4.7; Table 1). There was no pattern observed between storage time and Cq increase in swab samples. Using the test results on stool as the reference, the sensitivity of swab testing was Cq value dependent: swabs were positive for 91% of the results where the corresponding Cq on stool was <25 (range from 71% for Giardia to 100% for multiple targets), however this fell to 80% when the corresponding Cq on stool was < 30. The correlation of Cq values between stool and swab ranged from good for astrovirus (Pearson Coefficient R^2^ = 0.83) to no correlation for enterovirus (R^2^ = 0), as shown in Table 1.

**Table 1:** Comparison of enteropathogen detection on 129 stool and swab sample pairs collected on the same day. Sensitivity and specificity of detection on swabs was calculated using the results from the corresponding stool as the reference.

	Stool positive, Cq<25			Stool positive, Cq<30			Stool positive, Cq<35			Stool negative			Cq correlation (both Cqs < 35)		Average Cq (both Cqs < 35)		
	Swab +	swab -	Sensitivity, %	Swab +	swab -	Sensitivity, %	Swab +	swab -	Sensitivity, %	Swab+, Cq<25	Swab+, Cq<35	Specificity, %	R^2^	P	Swab	Stool	P *
**Astrovirus**	4	0	100	5	1	83	6	4	60	0	2	98	0.834	0.011	21.4±8.3	21.2±7.8	0.600
**Enterovirus**	4	1	80	15	9	63	22	21	51	0	2	98	0.000	0.968	31.3±2.3	28.1±3.0	**0.002**
**Norovirus GII**	8	1	89	10	4	71	10	7	59	0	0	100	0.333	0.081	26.9±3.3	23.6±3.1	**0.007**
**Rotavirus**	16	0	100	31	5	86	34	8	81	0	2	98	0.217	0.006	29.7±3.5	25.3±2.9	**<0.001**
**Sapovirus**	3	0	100	6	3	67	10	14	42	0	1	99	0.557	0.013	30.3±4.1	27.1±4.6	**0.022**
**Adenovirus**	17	1	94	21	6	78	34	18	65	0	2	97	0.751	<0.001	27.7±6.6	23.4±8.0	**<0.001**
***B*. *fragilis***	1	0	100	2	1	67	4	7	36	0	4	97	0.051	0.715	31.2±3.6	29.0±4.2	0.345
***Campylobacter***	13	2	87	19	6	76	24	20	55	1	9	89	0.102	0.129	29.2±4.1	25.8±4.2	**0.004**
***Cryptosporidium***	14	0	100	17	2	89	19	11	63	0	4	96	0.782	<0.001	25.5±5.2	22.6±5.2	**<0.001**
**EAEC**^#^	49	5	91	64	13	83	73	23	76	3	12	64	0.090	0.010	26.3±5.2	22.9±5.2	**<0.001**
**EPEC**^#^	20	2	91	30	10	75	39	28	58	0	3	95	0.259	0.001	29.0±4.8	24.4±5.4	**<0.001**
**ETEC**^#^	43	5	90	50	8	86	57	20	74	0	7	87	0.391	<0.001	26.7±5.1	21.2±5.8	**<0.001**
***E*. *bieneusi***	2	0	100	5	3	63	8	11	42	0	3	97	0.544	0.037	30.4±3.2	28.5±4.1	0.069
***Giardia***	5	2	71	8	3	73	9	11	45	0	1	99	0.171	0.270	30.2±3.0	25.2±3.6	**0.008**
***Salmonella enterica***	0	1	0	2	2	50	3	2	60	0	1	99	0.792	0.301	27.8±4.2	29.9±3.6	1.000
***Shigella*/EIEC**	12	1	92	18	2	90	21	10	68	0	2	98	0.584	<0.001	28.1±4.4	23.7±5.1	**<0.001**
**Total**	211	21	91	303	78	80	373	215	63	4	55	96	-	-	28.0±5.1	24.0±5.5	**<0.001**

* Wilcoxon Signed Ranks Test.

^#^ EAEC: enteroaggregative *E*. *coli*, aaiC or aatA; EPEC: enteropathogenic *E*. *coli*, eae with or without bfpA; ETEC: enterotoxigenic *E*. *coli*, LT or STh or STp.

### Total nucleic acid extraction using QIAamp Stool DNA Mini kit

Nucleic acid extracted with QIAamp Stool DNA Mini kit (“DNA alone”) was compared with a 1:1 mixture of DNA and RNA extracts (“DNA+RNA”). Paired testing on 41 samples was performed. These samples were chosen because they enabled evaluation of a range of pathogens, including adenovirus, astrovirus, enterovirus, norovirus GI and GII, rotavirus, sapovirus, enteroaggregative *E*. *coli*, enteropathogenic *E*. *coli*, enterotoxigenic *E*. *coli*, shiga-toxin producing *E*. *coli*, *Shigella*/enteroinvasive *E*. *coli*, *Aeromonas* spp., *B*. *fragilis*, *C*. *difficile*, *Campylobacter* spp., *H*. *pylori*, *M*. *tuberculosis*, *Salmonella enterica*., *V*. *cholerae*, *Cryptosporidium* spp., *C*. *cayetanensis*, *E*. *bieneusi*, *Giardia* spp., *S*. *stercoralis*. All the samples yielded positive results for more than one target. There were 318 matching positive results in both extracts, 17 positive results only in the DNA alone extracts, and 26 positive results only in the DNA+RNA extracts. These 43 discrepant results exclusively occurred at higher Cqs (33.5 ± 1.0 for DNA+RNA, 33.8 ± 0.9 for DNA alone, versus 25.7 ± 5.9 for the concordant results, *P* < 0.05), and were not concentrated among any particular pathogens. There was no difference between Cqs of DNA alone and DNA+RNA on all targets (ΔCq_(DNA+RNA-DNA)_ = -0.01 ± 1.17, *P* = 0.972, N = 318). Additionally, the linear correlation showed excellent Pearson coefficient (R^2^ = 0.9611). Among all RNA targets evaluated, only norovirus GII revealed a small decrement in Cq (ΔCq = -0.90 ± 1.26, *P* = 0.008, N = 18, Table 2). Thus the QIAamp Stool DNA Mini kit method allowed sufficient detection of not only DNA but also RNA targets. Of note, RNA extraction alone yielded similar viral detections to DNA alone or DNA+RNA (ΔCq = 0.3 ± 0.9, *P* = 0.356, comparing Cq for adenovirus, astrovirus, norovirus, sapovirus from 7 stool samples), but diminished bacterial detections presumably due to its use of saline supernatant from stool suspension (ΔCq = 8.4 ± 2.3, *P* < 0.05 for *Campylobacter*, EAEC, EPEC, ETEC, and *Cryptosporidium*). An alternative QIAamp Fast Stool DNA Mini kit operated both manually and by QIAcube with the Human DNA Analysis Protocol was also evaluated. Overall among all targets there was no statistically significant difference in Cq (26.5 ± 5.6 with the original QIAamp Stool DNA Mini kit vs. 26.4 ± 5.7 with the QIAamp Fast Stool DNA Mini kit, *P* = 0.261). Differences when they occurred were typically in the setting of low quantity detections.

**Table 2:** Comparison of total nucleic acid extract versus 1:1 mixed DNA and RNA extracts. For the RNA derived targets below, we compared Cqs between nucleic acid extracted with QIAamp Stool DNA mini kit versus a 1:1 mixture of QIAamp stool DNA mini kit extract and FUJIfilm QuickGene RNA tissue kit or QIAamp viral RNA mini kit extract.

	Average ΔCq [Cq_(DNA+RNA)_−Cq_(DNA alone)_]	ΔCq SD	*P* value	N
**Astrovirus**	0.20	1.11	0.516	14
**Enterovirus**	-0.27	1.00	0.325	14
**Norovirus GI**	-0.42	1.23	0.340	9
**Norovirus GII**	-0.90	1.26	**0.008**	18
**Rotavirus**	-0.20	1.47	0.634	13
**Sapovirus**	-0.19	1.69	0.837	4
***Cryptosporidium***	-0.38	0.81	0.261	7
***Giardia***	-0.55	2.30	0.369	15

### Expanded TAC panel for detection of additional enteropathogens

We previously included the most common 19 diarrheagenic pathogens on our TAC card and described their quantitative association with diarrhea [4, 7]. In the course of this work we expanded the assays to include 42 additional pathogens or certain subtypes (S1 Table). The same validation scheme was carried out as previously described [4, 7]. All the assays detected were 100% specific for the intended targets using a broad specificity panel (S2 Table). Analytical performance, including linearity, precision (data not shown), and limit of detection, was measured for each of these assays (S1 Table). For clinical sensitivity and specificity, at least 5 positive clinical samples and 20 negative samples were re-tested with confirmatory assays followed by amplicon sequencing. For the targets where no positive clinical sample was available, five spiked stool samples were included instead. As shown in Table 3, all of the assays exhibited 95–100% specificity except for the initial assay selected for detection of *Cyclospora cayetanensis* targeting the 18S rRNA gene region that was highly homologous to *Cystoisospora belli*. 98.2% (433/441) of the positive results were confirmed by amplicon sequencing. Results that could not be confirmed with amplicon sequencing were from samples that yielded higher Cqs (33.4 ± 1.8, versus 27.4 ± 5.6 for the confirmed results, *P* < 0.001).

**Table 3:** Validation of qPCR assays on clinical samples, using confirmatory PCR assays and amplicon sequencing. The assays are listed in S1 Table.

Target	No. TAC positive		Confirmatory assay		No. TAC negative	Confirmatory assay negative	Sensitivity vs. sequencing^#^	Specificity
	Clinical	Spiked	No. positive*	Sequencing*				
**Adenovirus C**	-	5	5	-	20	20	100%	100%
**Adenovirus F**	15	-	15	15	30	30	100%	100%
***Aeromonas***	15	-	15	15	30	30	100%	100%
***Ancylostoma duodenale***	3	5	8	3	30	30	100%	100%
***Ascaris lumbricoides***	8	-	8	8	30	30	100%	100%
***Bacteroides fragilis***	15	-	14	14	30	31	100%	97%
***Blastocystis***	7	-	7	6	20	21	100%	95%
***Clostridium difficile*_tcdA**	6	-	6	6	30	30	100%	100%
***Campylobacter coli***	9	-	9	9	20	20	100%	100%
***Campylobacter jejuni***	15	-	15	15	20	20	100%	100%
***Campylobacter* spp**	15	-	15	15	30	30	100%	100%
***Cryptosporidium hominis***	15	-	15	15	30	30	100%	100%
***Cryptosporidium parvum***	15	-	15	15	30	30	100%	100%
***Cyclospora cayetanensis* (1)**	45	-	14	13	32	64	100%	50%
***Cyclospora cayetanensis* (2)**	15	-	15	15	30	30	100%	100%
***Cystoisospora belli***	13	-	12	12	30	31	100%	97%
**Cytomegalovirus**	15	-	15	14	20	21	100%	95%
**Ebola virus**	-	5	5	-	20	20	100%	100%
***Enterocytozoon bieneusi***	15	-	15	15	30	30	100%	100%
***Entamoeba* spp**	9	-	9	9	20	20	100%	100%
***Encephalitozoon intestinalis***	3	5	8	3	30	30	100%	100%
**Enterovirus**	15	-	15	15	30	30	100%	100%
**EAEC_aar**	15	-	15	15	30	30	100%	100%
**EAEC_aggR**	15	-	15	15	30	30	100%	100%
***Escherichia coli* O157**	2	5	7	2	20	20	100%	100%
**Epstein-Barr virus**	-	5	5	-	20	20	100%	100%
***Giardia* assemblage A**	15	-	15	15	30	30	100%	100%
***Giardia* assemblage B**	15	-	15	15	30	30	100%	100%
***Helicobacter pylori***	15	-	15	15	30	30	100%	100%
**Human herpesvirus 6**	6	-	6	6	20	20	100%	100%
**Human herpesvirus 7**	-	5	5	-	20	20	100%	100%
***Listeria monocytogenes***	-	5	5	-	20	20	100%	100%
***Mycobacterium tuberculosis***	15	-	14	14	30	31	100%	97%
***Necator americanus***	3	5	8	3	30	30	100%	100%
**Norovirus GI**	15	-	15	15	30	30	100%	100%
**Norovirus GI.1**	12	3	12	12	20	20	100%	100%
**Norovirus GII.4**	15	-	15	15	20	20	100%	100%
***Plesiomonas shigelloides***	10	-	10	9	20	21	100%	95%
**Rotarix**	-	5	5	-	20	20	100%	100%
**RotaTeq**	-	5	5	-	20	20	100%	100%
***Salmonella* invA**	8	-	8	8	20	20	100%	100%
***Salmonella* ompC**	8	-	8	8	20	20	100%	100%
***Salmonella* ttr**	15	-	15	15	30	30	100%	100%
***Schistosoma mansoni***	2	5	7	2	20	20	100%	100%
***Strongyloides stercoralis***	3	5	8	3	30	30	100%	100%
***Vibrio cholerae***	12	-	12	12	30	30	100%	100%
***Vibrio parahaemolyticus***	2	5	7	2	30	30	100%	100%
***Yersinia***	-	5	5	-	30	30	100%	100%

* No. positive included both clinical and spiked samples, but only positive clinical samples were subjected to amplicon sequencing.

^#^ The sensitivity was calculated using sequencing as the reference for most of the targets. When only spiked samples were tested, the sensitivity was based on the results of confirmatory assays.

### Quantification of pathogen target copy number by qPCR

**Fig 1:**
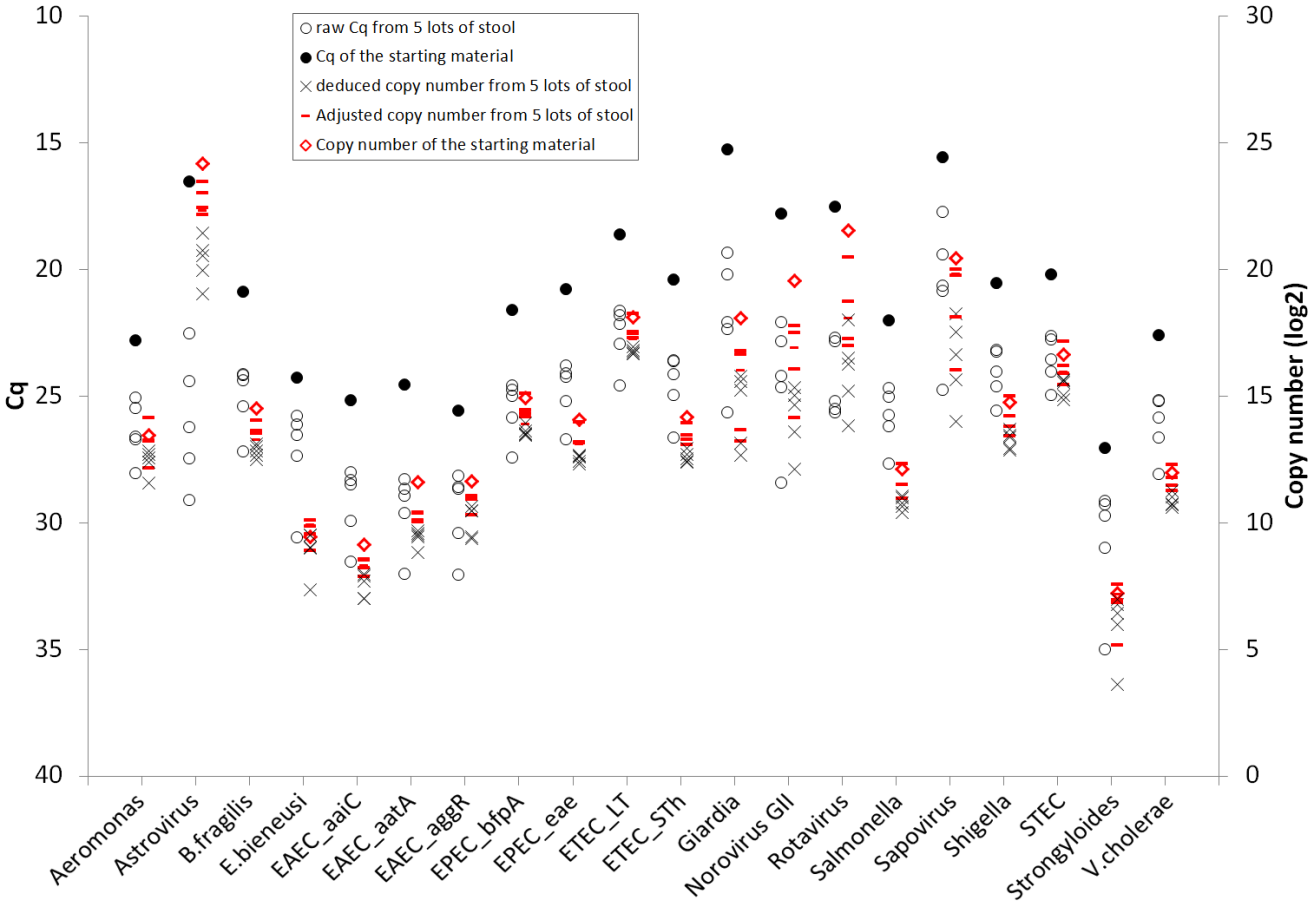
Demonstration of quantification on spiked analytical samples. Five lots of stool with different amount of inhibitors were prepared, then the mixture of target nucleic acid was spiked. Extraction and testing with TAC were performed, target copy numbers were calculated based on standard curves and normalized to extrinsic controls. Target copy numbers (circles) were log2 transformed in order to be on the same scale as Cq.

Since all assays exhibited good linearity we proceeded to generate standard curves in order to transform Cq data into copy numbers, including adjusting for the extraction and amplification efficiency of the specimen. Specifically, an identical amount of pathogen nucleic acid was spiked into five different lots of stool that exhibited different extraction and amplification efficiencies (ranged from 0.1% to 40% as measured by MS2 and PhHV). The distribution of the raw Cqs in the 5 specimens, the deduced copy numbers from the Cqs, and copy numbers after adjustment to each specimen’s extraction and amplification efficiency are shown in Fig 1. The raw Cqs for each target in the 5 specimens differed by 2.8 ± 1.6 for *E*. *bieneusi* and up to 9.3 ± 2.3 for astrovirus (Fig 1; overall 4.4 ± 2.1, approximately 20 fold). The deduced copy numbers therefore differed substantially as well. However, after adjustment for the specimen’s individual efficiency, the adjusted copy numbers were tighter and more accurately estimated the amount of starting material, within 2 fold (on average the estimate was 59.3 ± 31.9% of the starting material compared with 8.5 ± 6.8% without adjustment).

## Discussion

Detection of enteropathogens has been greatly advanced with molecular diagnostics. This work was of a practical nature, to optimize relevant steps for purposes of throughput and quantification. The advantages of using rectal swabs include the instant ability to obtain a specimen with fewer demands on specimen transportation and storage. We did not observe loss of signal over a 3 week time period at room temperature, further simplifying collection and transport. As has been previously noted [1], rectal swabs did yield higher Cqs than stool, but still detected pathogens when reasonably abundant (80% sensitivity for stool with detection of pathogens at Cq<30). The sensitivity dropped to sixties when including the low level detections (Cq<35). For unclear reasons enterovirus was particularly poorly detected in swabs (71% of enterovirus positive stool yielded Cqs 2–12 lower than those of the corresponding swabs). Such discrepancies in Cq between stool and swab could partly be explained by the smaller amount of specimen recovered from swabs. For example, using 16S rRNA gene as an indicator of feces mass, 200 mg of stool did yield lower Cqs than swab (average 17.6±7.2 vs. 20.6±7.2, Wilcoxon Signed Ranks Test *P*<0.001). Distinct locations of infections (e.g., lumenal vs. mucosal) could also play a role. Overall, however, we feel swabs are a reasonable and convenient alternative for molecular identification of enteropathogens when stool is not available.

Typically DNA is extracted from stool for amplification of bacteria and parasites, while RNA is extracted for the many RNA viruses. RNA is known to be labile to high temperatures, yet even with the 95°C incubation step that was essential to improve DNA yield, we found that RNA was isolated with the QIAamp Stool DNA Mini or Fast Stool DNA Mini kits. Furthermore we found RNA can be isolated from the whole stool sample as opposed to the stool supernatant used with most viral RNA extraction protocols [9], with a comparable RNA yield. Cq values for norovirus GII were significantly higher with the single extraction, however the difference was within 1 Cq unit, which should be acceptable for most purposes [4]. Rotavirus is a double stranded RNA virus and requires an extra pre-denaturation step or thermostable reverse transcriptase in order to obtain efficient amplification [10]. Presumably due to the 5 minute incubation at 95°C in our extraction procedure and high nucleic acid complexity in the samples, we again found that the QIAamp Stool DNA extracts can be directly subjected to RT-PCR with regular enzyme mix to yield similar rotavirus detection (data not shown). We initially supplemented the QIAamp Stool DNA extracts with RNA storage solution to maintain the RNA stability but found that RNA targets were consistently detected over six months of storage at -80°C without this supplement (data not shown). Total nucleic acid extraction saves specimen, reduces bench time and reagent cost, and simplifies procurement and shipment for field studies (since some viral extraction kits contain hazardous materials).

In this work we share a comprehensive list of TaqMan probe based qPCR assays by adding another 42 pathogens that could be present in the gastrointestinal tract. We expanded the list due to the availability of space on the TaqMan Array Card platform since we have found that results are highly reproducible, such that single reactions are sufficient and duplicates are not required, particularly at the high quantities when diarrhea occurs. We thus find quantification to be advantageous compared to other qualitative multiplex panels, both in resource-limited settings where pathogens are highly prevalent in asymptomatic controls [5, 11, 12] and possibly even in developed countries [13–15]. We and others have shown that for many enteropathogens the association with disease is quantity dependent. This is usually reported as quantification cycles [4, 16–18], however reporting copy numbers of each pathogen may be preferable because it is qPCR assay and platform-independent [19–21]. We therefore describe a procedure to generate standard curves using positive control constructs and then calculate pathogen load. This quantity can be further adjusted to the extraction/amplification efficiency of the specimen via the spiked extrinsic controls. Worth noting is that this yields the copy number of the gene target. This should equate to the pathogen quantity for the many gene targets that are single copy in the genome, such as most of the viral targets and some chromosomal bacterial targets (e.g., hlyA of *V*. *cholerae*, ttr of *Salmonella*, ureC of *H*. *pylori*, cadF of *Campylobacter*, aaiC of EAEC, eae of EPEC). However plasmid borne virulent factors (e.g., ipaH of *Shigella*/EIEC, ST and LT of ETEC, bfpA of EPEC) and ribosomal rRNA targets are variable and multiple copy targets, thus the copy number may exceed the pathogen quantity.

Taken together these improvements in specimen collection, nucleic acid extraction, and quantification should help expand the application of molecular diagnostics to enteric diseases.

## Supporting Information

S1 Table Primer and probe sequences for TaqMan-MGB probe based real time PCR assays used on TaqMan Array Card and their analytical performance. (PDF)

S2 Table List of genomic material included in the specificity testing of all the assays. (PDF)
