# Supplementary material for: Optimization of Quantitative PCR Methods for Enteropathogen Detection
Source: PLoS One. 2016 Jun 23;11(6):e0158199. doi: 10.1371/journal.pone.0158199 (PMC4918952; doi:10.1371/journal.pone.0158199)
Supplement: S1 Table — (PDF) [file pone.0158199.s001.pdf]

S1 Table. Primer and probe sequences for TaqMan-MGB probe based real time PCR assays used on TaqMan Array Card and their analytical performance.

	Pathogen	Gene	Strand	Sequence used on TAC	Reference	PCR efficiency, %	R^2	Limit of Detection (per gram of stool)
Virus	Adenovirus F	Fiber gene	forward	AACTTTCTCTCTTAATAGACGCC	Modified from [1]	94.5	0.999	10^5
			reverse	AGGGGGCTAGAAAACAAAA				
			probe	CTGACACGGGCACTCT				
	Adenovirus C	hexon	forward	GGACCGCATGTACTCSTTCTT	Modified from [2]	103.4	0.998	10^5
			reverse	TTTAGTATCRTCCACCACCTGACG				
			reverse	TTTAGTATCGTCCACCACTTGCC				
			probe	AGAAACTTCCAGCCCATGAGC				
	Adenovirus*	Hexon	forward	GCCACGGTGGGGTTTCTAAACTT	[3]	98.2	0.999	10^5
			reverse	GCCCCAGTGGTCTTACATGCACATC				
			probe	TGCACCAGACCCGGGCTCAG				
	Astrovirus*	Capsid	forward	CAGTTGCTTGCTGCGTTCA	[4]	95.9	0.992	10^6
			reverse	CTTGCTAGCCATCACACTTCT				
			probe	CACAGAAGAGCAACTCCATCGC				
	Cytomegalovirus	glycoprotein B	forward	AGGTCTTCAAGGAACTCAGCAAGA	Modified from [5]	91.5	0.999	10^5
			reverse	CGGCAATCGGTTTGTTGTAAA				
			probe	AMCCCGTCAGCCATTCTCTCGGC				
		Immediate Early 2	forward	GAGCCCGACTTTACCATCCA	[6]			
			reverse	CAGCCGGCGGTATCGA				
			probe	ACCGCAACAAGATT				
	Ebola virus	NP40	forward	TGGAAAAAACATTAAGAGAACACTTGC	[7]	93.1	0.991	10^6
			reverse	AGGAGAGAAACTGACCGGCAT				
			probe	CATGCCGGAAGAGGAGACAACTGAAGC				
	Enterovirus	5’UTR	forward	CCCTGAATGCGGCTAATCC	Modified from [8]	92.0	0.995	10^6
			reverse	GCGATTGTCACCATWAGCAG				
			probe	CCGACTACTTTGGGWGTCCGT				
	Epstein-Barr virus	DNA polymerase	forward	CGGAAGCCCTCTRGACTTC	Modified from [9]	96.8	1.000	10^5
			reverse	CCCTGTTTATCCGATGGAATG				
			probe	FAM-TGTACACGCACGAGAAATGCG				
	Human Herpesvirus 6	U38	forward	CCAGTCAGACAGTTGTTTCGG	This work	94.8	0.999	10^5
			reverse	GGCCGCATTCGTACAGATAC				
			probe	CAGTAAGACGGGATATAATGCC				
	Human Herpesvirus 7	U10-11	forward	TGGTGTCAAGCTATCCTAATGAA	This work	98.4	0.999	10^5
			reverse	GAGGAGAATTCTGTACCCATGG				
			probe	CACATTTGTACTTCAAAGTAGCC				
	Norovirus GI	ORF1-2	forward	CGYTGGATGCGNTTYCATGA	Modified from [10]	93.6	0.997	10^6
			reverse	CTTAGACGCCATCATCATTYAC				
			probe	TGGACAGGAGATCGC				
	Norovirus GI.1	ORF1-2	forward	AGGATCCATTGCAAGAGGG	This study	89.3	0.992	10^6
			reverse	CTACATCAAGCGTGGATGGC				
			probe	TGGTCAGTTGGTACCGGAG				
	Norovirus GII*	ORF1-2	forward	CARGARBCNATGTTYAGRTGGATGAG	[10]	94.9	0.991	10^6
			reverse	TCGACGCCATCTTCATTCACA				
			probe	TGGGAGGGCGATCGCAATCT				
	Norovirus GII.4	ORF1-2	forward	GARTGACGCCARCCCATCTG	This study	91.0	0.991	10^6

			reverse	ATCCAGGGGTCAATTAYATTTTGT				
			probe	CCTCTGGGACRAGGTTGGCT				
	Rotavirus*	NSP3	forward	ACCATCTWCACRTRACCCTCTATGAG	[11]	94.6	0.997	10^6
			reverse	GGTCACATAACGCCCCTATAGC				
			probe	AGTTAAAAGCTAACACTGTCAAA				
	Rotarix	NSP2	forward	CTAACCATGCGGATAGAGTGTTC	Modified from [12]	88.3	0.986	10^6
			reverse	TTGAAGAC GT AAATGCA TAC CAA TTC				
			probe	TCCAATAGATTGAAGTCAGTAACG				
	RotaTeq	VP6	forward	GCGGCGTTATTTCCAAATGCACAG	[12]	93.1	0.991	10^6
			reverse	CGTCGGCAA GCAC TGATTCA CAAA				
			probe	ATCACGCAACAGTAGGACTCACGCTT				
	Sapovirus*	RdRp	forward	GAYCASGCTCTCGCYACCTAC	[4]	98.0	0.985	10^6
			forward	TTGGCCCTCGCCACCTAC				
			reverse	CCCTCCATYTCAAACACTA				
			probe	CCRCCTATRAACCA				
Bacteria	EAEC	aaiC*	forward	ATTGTCCTCAGGCATTTCAC	[13]	99.7	0.992	10^5
			reverse	ACGACACCCCTGATAAACAA				
			probe	TAGTGCATACTCATCATTTAAG				
		aatA*	forward	CTGGCGAAAGACTGTATCAT	[13]	91.7	0.991	10^5
			reverse	TTTTGCTTCATAAGCCGATAGA				
			probe	TGGTTCTCATCTATTACAGACAGC				
		aar	forward	AGCTCTGGAAACTGGCCTCT	Modified from [14]	100.9	0.997	10^5
			reverse	AACCGTCCTGATTTCTGCTT				
			probe	CCTCGCAAAACATTGCTCTA				
		aggR	forward	GCAATCAGATTAARCAGCGATACA	[14]	100.8	0.999	10^5
			reverse	TTCGGACAACTRCAAGCATC				
			probe	AAGACGCCTAAAGGATGCCC				
	EPEC	eae*	forward	CATTGATCAGGATTTTTCTGGTGATA	[4]	99.8	0.999	10^5
			reverse	CTCATGCGGAAATAGCCGTTA				
			probe	ATACTGGCGAGACTATTTCAA				
		bfpA	forward	TGGTGCTTGCGCTTGCT	[4]	95.7	0.999	10^5
			reverse	CGTTGCGCTCATTACTTCTG				
			probe	CAGTCTGCGTCTGATTCCAA				
	ETEC*	LT*	forward	TTCCCACCGGATCACCAA	[15]	93.0	0.999	10^5
			reverse	CAACCTTGTGGTGCATGATGA				
			probe	CTTGGAGAGAAGAACCCT				
		STh*	forward	GCTAAACCAGYAGRGTCTTCAAAA	[4]	95.9	0.999	10^5
			forward	CCCGGTACARGCAGGATTACAACA				
			reverse	TGGTCCTGAAAGCATGAA				
		STp*	reverse	TGAATCACTTGACTCTTCAAAA	[4]	96.5	0.997	10^5
			probe	GGCAGGATTACAACAAAGTT				
			probe	TGAACAACACATTTTACTGCT				
	STEC	*stx1**	forward	ACTTCTCGACTGCAAAGACGTATG	[4]	104.5	0.998	10^5
			reverse	ACAAATTATCCCCTGWGCCACTATC				
			probe	CTCTGCAATAGGTACTCCA				
		*stx2**	forward	CCACATCGGTGTCTGTTATTAACC	[15]	105.2	0.994	10^5
			reverse	GGTCAAAACGCGCCTGATAG				
			probe	TTGCTGTGGATATACGAGG				
	E. coli O157	*rfbE*	forward	TTTCACACTTATTGGATGGTCTCAA	[16]	96.7	0.994	10^5
			reverse	CGATGAGTTTATCTGCAAGGTGAT				

		probe	CTCTCTTTCCTCTGCGGTCCT				
Aeromonas	Aerolysin	forward	TYCGYTACCAGTGGGACAAG	[4]	97.7	0.996	10^5
		reverse	CCRGCAAACTGGCTCTCG				
		probe	CAGTTCCAGTCCCACCACTT				
Bacteroides fragilis	EGBF	forward	GGGACAAGGATTCTACCAGCTTTATA	Modified from [17]	99.7	0.994	10^5
		reverse	ATTCGGCAATCTCATTCATCATT				
		probe	CAATGGCGAATCCATCAG				
*C. jejuni/C. coli**	cadF	forward	CTGCTAAACCATAGAAATAAAATTTCTCAC	[18]	97.3	0.999	10^5
		reverse	CTTTGAAGGTAATTTAGATATGGATAATCG				
		probe	CATTTTGACGATTTTTGGCTTGA				
Campylobacter spp.	Cpn60	forward	AAAGTIGGMAAAGATGGTGTTAT	Designed based on [19]	95.3	0.991	10^5
		forward	AAAGTIGGWAAAGACGGYGTTAT				
		reverse	TCAAATTGCATACCYTCAAC				
		probe	TTTGCCTCTTCMACAGT				
		probe	TTTGCTTCTTCWACAGT				
Campylobacter jejuni	hipO	forward	CTTGCGGTCATGATGGACATAC	This wrok	97.5	1.000	10^5
		reverse	AGCACCACCCAAACCCTCTTCA				
		probe	TGCTTGCTGCAAAGTATT				
Campylobacter coli	GlyA	forward	AAACCAAAGCTTATCGTGTGC	This work	96.2	0.995	10^5
		reverse	AGTGCAGCAATGTGTGCAAT				
		probe	TAAGCTCCAACTTCATCCG				
Clostridium difficile	tcdB*	forward	GGTATTACCTAATGCTCCAAATAG	[4]	97.2	0.992	10^5
		reverse	TTTGTGCCATCATTTTCTAAGC				
		probe	CCTGGTGTCCATCCTGTTTC				
	tcdA	forward	TTCAAGCAGAAATAGAGCACTC	[4]	96.3	0.990	10^5
		reverse	TATCAGCCCATTGTTTTATGTATTC				
		probe	CACTGACTTCTCCACCTATCCA				
Helicobacter pylori	ureC	forward	GACACCAGAAAAAGCGGCTA	Designed based on [20]	101.6	0.997	10^5
		reverse	AGCGCATGTCTTCGGTTAAA				
		probe	TCACTAAAGCGTTTTCTACC				
Listeria monocytogenes	hly	forward	TTTCATCCATGGCACCACC	This work	96.7	0.997	10^5
		reverse	ATCCGCGTGTTTCTTTTCGA				
		probe	CGCCTGCAAGTCCTAAGACGCCA				
Mycobacterium tuberculosis	IS6110	forward	GGGTAGCAGACCTCACCTATG	[21]	95.3	0.994	10^5
		reverse	AGCGTAGGCGTCGGTGA				
		probe	TCGCCTACGTGGCCTTT				
Plesiomonas shigelloides	*gyrB*	forward	CCGCCGTGAAGGCAAAG	This work	96.3	0.999	10^5
		reverse	GCTACCGGCTCACCCAGAT				
		probe	CACACCCAAGAATAC				
Salmonella enterica	*ttr*	forward	CTCACCAGGAGATTACAACATGG	[22]	98.6	1.000	10^5
		reverse	AGCTCAGACCAAAAGTGACCATC				
		probe	CACCGACGGCGAGACCGACTTT				
	ompC	forward	ACCGCTAACGCTCGCCTGTAT	This work	96.4	0.994	10^5
			ACCGCTGACGAACACCTGTAT				
		reverse	CGGGTTGCGTTATAGGTCTGA				
		probe	AATACTGCGCTGCCAGAT				
	invA	forward	GGCAATTCGTTATTGGCGATA	This work	94.1	0.996	10^5
		reverse	CACGGTGACAATAGAGAAGACAACA				
		probe	CCTGGCGGTGGGTT				
Salmonella enterica	STY0201	forward	CGCGAAGTCAGAGTCGACATAG	[23]	93.5	0.994	10^5

	serovar Typhi		reverse	AAGACCTCAACGCCGATCAC				
			probe	CAGCCTGCTCCAGAACA				
	Shigella/EIEC*	*ipaH*	forward	CCTTTTCCGCGTTCCTTGA	[24]	99.2	0.998	10^5
			reverse	CGGAATCCGGAGGTATTGC				
			probe	CGCCTTTCCGATACCGTCTCTGCA				
	Vibrio cholerae	*hlyA*	forward	ATCGTCAGTTTGGAGCCAGT	Modified from [4]	99.7	0.997	10^5
			reverse	TCGATGCGTTAAACACGAAG				
			probe	ACCGATGCGATTGCCCAA				
	Vibrio parahaemolyticus	*toxR*	forward	GTTTGGCGTGAGCAAGGTTT	Modified from [25]	96.1	0.998	10^5
			reverse	AAGCGGGCTTAGGCGTTCA				
			probe	TCAAGCGATTTCTACTCTGCG				
	Yersinia enterocolitica	*lytA*	forward	TGATTCACCAGCAGCAATAC	[25]	93.1	0.994	10^5
			reverse	GGCATCATGAAAGGCGG				
			probe	TGTCGGTTTCTCCTTCCAGG				
Fungi	*Encephalitozoon intestinalis*	SSU rRNA	forward	TGTGTAGGCGTGAGAGTGTATCTG	[26]	98.0	0.998	10^5
			reverse	CATCCAACCATCACGTACCAATC				
			probe	CACTGCACCCACATCCCTCACCCTT				
	Enterocytozoon bieneusi	*ITS*	forward	CACCAGGTTGATTCTGCCTGAC	[26]	101.4	1.000	10^5
			reverse	CTAGTTAGGCCATTACCCTAACTACCA				
			probe	CTATCACTGAGCCGTCC				
Protozoa	*Cryptosporidium* spp.*	18S rRNA	forward	GGGTTGTATTTATTAGATAAAGAACCA	[4]	93.2	0.994	10^5
			reverse	AGGCCAATACCCTACCGTCT				
			probe	TGACATATCATTCAAGTTTCTGAC				
	Cryptosporidium hominis	*LIB13*	forward	TCCTTGAAATGAATATTTGTGACTCG	[27]	94.1	0.996	10^5
			reverse	AAATGTGGTAGTTGCGGTTGAAA				
			probe	CTTACTTCGTGGCGGCGT				
	Cryptosporidium parvum	*LIB13*	forward	TCCTTGAAATGAATATTTGTGACTCG	[27]	92.6	0.992	10^5
			reverse	TTAATGTGGTAGTTGCGGTTGAAC				
			probe	TATCTCTTCGTAGCGGCGTA				
	*Entamoeba histolytica**	18S rRNA	forward	ATTGTCGTGGCATCCTAACTCA	[28]	95.2	0.989	10^5
			reverse	GCGGACGGCTCATTATAACA				
			probe	TCATTGAATGAATTGGCCATTT				
	Entamoeba spp.	18S rRNA	forward	AAACGATGTCAACCAAGGATTG	This work	91.0	0.994	10^5
			reverse	TCCCCCTGAAGTCCATAAACTC				
			probe	CCTTGTTCAGAACTTAAAGAGAAA				
	Giardia spp.*	18S rRNA	forward	GACGGCTCAGGACAACGGTT	[28]	99.6	0.999	10^5
			reverse	TTGCCAGCGGTGTCCG				
			probe	CCCGCGGCGGTCCCTGCTAG				
	Giardia assemblage A	triosephosphate	forward	TTCCGCCGTACACCTGTC	Modified from [29]	94.6	0.986	10^5
		isomerase (TPI)	reverse	GCGCTGCTATCCTCAACTG				
			probe	ATTGCGGCAAACACGTCA				
	Giardia assemblage B	triosephosphate	forward	GATGAACGCAAGGCCAATAA	This work	96.7	0.994	10^5
		isomerase (TPI)	reverse	CTTTGATTCTCCAATCTCCTTCTT				
			probe	AATATTGCTCAGCTCGAGGC				
	Cyclospora cayetanensis¶	18S rRNA (1)	forward	TAGTAACCGAACGGATCGCATT	[30]	98.1	0.999	10^5
			reverse	AATGCCACGGTAGGCCAATA				
			probe	CCGGCGATAGATCATTCAAGTTTCTGACC				
		18S rRNA (2)	forward	AAAAGCTCGTAGTTGGATTTCTG	Modified from (29)			
			reverse	AACACCAACGCACGCAGC				
			probe	AAGGCCGGATGACCACGA				

	Cystoisospora belli	18S rRNA	forward	ATATTCCCTGCAGCATGTCTGTTT	[31]	101.8	0.999	10^5
			reverse	CCACACGCGTATTCCAGAGA				
			probe	CAAGTTCTGCTCACGCGCTTCTGG				
Helminth	*Ancylostoma duodenale*	*ITS2*	forward	GAATGACAGCAAACTCGTTGTTG	[32]	97.1	0.997	10^5
			reverse	ATACTAGCCACTGCCGAAACGT				
			probe	ATCGTTTACCGACTTTAG				
	Blastocystis spp.	18S rRNA	forward	TGGTCCGRTGAACACTTTGGAT	This work	93.1	0.999	10^5
			reverse	CCTACGGAAACCTTGTTACGACTTCA				
			probe	CTTCCTCTAAATGRTAAGATT				
	Necator americanus	*ITS2*	forward	CTGTTTGTCGAACGGTACTTGC	[32]	96.7	0.997	10^5
			reverse	ATAACAGCGTGCACATGTTGC				
			probe	CTGTACTACGCATTGTATAC				
	Ascaris lumbricoides	*ITS1*	forward	GCCACATAGTAAATTGCACACAAAT	Modified from [33]	97.5	0.999	10^5
			reverse	GCCTTTCTAACAAGCCCAACAT				
			probe	TTGGCGGACAATTGCATGCGAT				
	Strongyloides stercoralis	Dispersed	forward	TCCAGAAAAGTCTTCACTCTCCAG	[34]	99.1	0.986	10^5
		repetitive	reverse	TGCGTTAGAATTTAGATATTATTGTTGCT				
		sequence	probe	TCAGCTCCAGTTGAACAACAGCCTCCAA				
	Schistosoma mansoni		forward	GGTCTAGATGACTTGATYGAGATGCT	[35]	92.1	0.991	10^5
			reverse	TCCCGAGCGYGTATAATGTCATTA				
			probe	TGGGTTGTGCTCGAGTCGTGGC				
	*Trichuris trichiura**	18S rRNA	forward	TTGAAACGACTTGCTCATCAACTT	[4]	97.4	0.997	10^5
			reverse	CTGATTCTCCGTTAACCGTTGTC				
			probe	CGATGGTACGCTACGTGCTTACCATGG				
Control	MS2*	*MS2g1*	forward	TGGCACTACCCCTCTCCGTATTCAC	[36]	95.1	0.996	-
			reverse	GTACGGGCGACCCCACGATGAC				
			probe	CACATCGATAGATCAAGGTGCCTACAAGC				
	PhHV*	*gB*	forward	GGGCGAATCACAGATTGAATC	[4]	94.4	0.997	-
			reverse	GCGGTTCCAAACGTACCAA				
			probe	TATGTGTCCGCCACCATCT				
	bacterial 16s		forward	TGCAAGTCGAACGAAGCACTTTA	[37]	93.4	0.994	-
			reverse	GCAGGTTACCCACGCGTTAC				
			probe	CGCCACTCAGTCACAAA				

* Targets included in the previous version of TAC [4,38].

¶ The initial assay (1) for *Cyclospora cayetanensis* adapted from [30] was found to detect *Cytoisosora belli* as well, which was later replaced with an alternative design (2) targeting the more diverse region of 18S gene capable of differentiating *Cyclospora* from *Cytoisosora*.

References

1. Jothikumar N, Cromeans TL, Hill VR, Lu X, Sobsey MD, et al. (2005) Quantitative real-time PCR assays for detection of human adenoviruses and identification of serotypes 40 and 41. Appl Environ Microbiol 71: 3131-3136.
2. Garnett CT, Erdman D, Xu W, Gooding LR (2002) Prevalence and quantitation of species C adenovirus DNA in human mucosal lymphocytes. J Virol 76: 10608-10616.
3. Heim A, Ebnet C, Harste G, Pring-Akerblom P (2003) Rapid and quantitative detection of human adenovirus DNA by real-time PCR. J Med Virol 70: 228-239.
4. Liu J, Gratz J, Amour C, Kibiki G, Becker S, et al. (2013) A laboratory-developed TaqMan Array Card for simultaneous detection of 19 enteropathogens. J Clin Microbiol 51: 472-480.
5. Boppana SB, Fowler KB, Pass RF, Rivera LB, Bradford RD, et al. (2005) Congenital cytomegalovirus infection: association between virus burden in infancy and hearing loss. J Pediatr 146: 817-823.
6. Boppana SB, Ross SA, Novak Z, Shimamura M, Tolan RW, Jr., et al. (2010) Dried blood spot real-time polymerase chain reaction assays to screen newborns for congenital cytomegalovirus infection. JAMA 303: 1375-1382.
7. Towner JS, Sealy TK, Ksiazek TG, Nichol ST (2007) High-throughput molecular detection of hemorrhagic fever virus threats with applications for outbreak settings. J Infect Dis 196 Suppl 2: S205-212.
8. Oberste MS, Penaranda S, Rogers SL, Henderson E, Nix WA (2010) Comparative evaluation of Taqman real-time PCR and semi-nested VP1 PCR for detection of enteroviruses in clinical specimens. J Clin Virol 49: 73-74.
9. Wadowsky RM, Laus S, Green M, Webber SA, Rowe D (2003) Measurement of Epstein-Barr virus DNA loads in whole blood and plasma by TaqMan PCR and in peripheral blood lymphocytes by competitive PCR. J Clin Microbiol 41: 5245-5249.
10. Kageyama T, Kojima S, Shinohara M, Uchida K, Fukushi S, et al. (2003) Broadly reactive and highly sensitive assay for Norwalk-like viruses based on real-time quantitative reverse transcription-PCR. J Clin Microbiol 41: 1548-1557.
11. Zeng SQ, Halkosalo A, Salminen M, Szakal ED, Puustinen L, et al. (2008) One-step quantitative RT-PCR for the detection of rotavirus in acute gastroenteritis. J Virol Methods 153: 238-240.
12. Gautam R, Esona MD, Mijatovic-Rustempasic S, Ian Tam K, Gentsch JR, et al. (2014) Real-time RT-PCR assays to differentiate wild-type group A rotavirus strains from Rotarix((R)) and RotaTeq((R)) vaccine strains in stool samples. Hum Vaccin Immunother 10: 767-777.
13. Boisen N, Struve C, Scheutz F, Krogfelt KA, Nataro JP (2008) New adhesin of enteroaggregative Escherichia coli related to the Afa/Dr/AAF family. Infect Immun 76: 3281-3292.
14. Boisen N, Scheutz F, Rasko DA, Redman JC, Persson S, et al. (2012) Genomic characterization of enteroaggregative Escherichia coli from children in Mali. J Infect Dis 205: 431-444.
15. Hidaka A, Hokyo T, Arikawa K, Fujihara S, Ogasawara J, et al. (2009) Multiplex real-time PCR for exhaustive detection of diarrhoeagenic Escherichia coli. J Appl Microbiol 106: 410-420.

16. Operario DJ, Moonah S, Houpt E (2014) Hemolytic uremic syndrome following infection with O111 Shiga toxin-producing Escherichia coli revealed through molecular diagnostics. J Clin Microbiol 52: 1003-1005.
17. Merino VR, Nakano V, Liu C, Song Y, Finegold SM, et al. (2011) Quantitative detection of enterotoxigenic Bacteroides fragilis subtypes isolated from children with and without diarrhea. J Clin Microbiol 49: 416-418.
18. Cunningham SA, Sloan LM, Nyre LM, Vetter EA, Mandrekar J, et al. (2010) Three-hour molecular detection of Campylobacter, Salmonella, Yersinia, and Shigella species in feces with accuracy as high as that of culture. J Clin Microbiol 48: 2929-2933.
19. Hill JE, Paccagnella A, Law K, Melito PL, Woodward DL, et al. (2006) Identification of Campylobacter spp. and discrimination from Helicobacter and Arcobacter spp. by direct sequencing of PCR-amplified cpn60 sequences and comparison to cpnDB, a chaperonin reference sequence database. J Med Microbiol 55: 393-399.
20. Shukla SK, Prasad KN, Tripathi A, Ghoshal UC, Krishnani N, et al. (2011) Quantitation of Helicobacter pylori ureC gene and its comparison with different diagnostic techniques and gastric histopathology. J Microbiol Methods 86: 231-237.
21. Halse TA, Edwards J, Cunningham PL, Wolfgang WJ, Dumas NB, et al. (2010) Combined real-time PCR and rpoB gene pyrosequencing for rapid identification of Mycobacterium tuberculosis and determination of rifampin resistance directly in clinical specimens. J Clin Microbiol 48: 1182-1188.
22. Malorny B, Paccassoni E, Fach P, Bunge C, Martin A, et al. (2004) Diagnostic real-time PCR for detection of Salmonella in food. Appl Environ Microbiol 70: 7046-7052.
23. Liu J, Ochieng C, Wiersma S, Stroher U, Towner JS, et al. (2016) Development of a TaqMan Array Card for Acute-Febrile-Illness Outbreak Investigation and Surveillance of Emerging Pathogens, Including Ebola Virus. J Clin Microbiol 54: 49-58.
24. Vu DT, Sethabutr O, Von Seidlein L, Tran VT, Do GC, et al. (2004) Detection of Shigella by a PCR assay targeting the ipaH gene suggests increased prevalence of shigellosis in Nha Trang, Vietnam. J Clin Microbiol 42: 2031-2035.
25. Liu J, Gratz J, Maro A, Kumburu H, Kibiki G, et al. (2012) Simultaneous detection of six diarrhea-causing bacterial pathogens with an in-house PCR-luminex assay. J Clin Microbiol 50: 98-103.
26. Verweij JJ, Ten Hove R, Brienen EA, van Lieshout L (2007) Multiplex detection of Enterocytozoon bieneusi and Encephalitozoon spp. in fecal samples using real-time PCR. Diagn Microbiol Infect Dis 57: 163-167.
27. Hadfield SJ, Robinson G, Elwin K, Chalmers RM (2011) Detection and differentiation of Cryptosporidium spp. in human clinical samples by use of real-time PCR. J Clin Microbiol 49: 918-924.
28. Verweij JJ, Blange RA, Templeton K, Schinkel J, Brienen EA, et al. (2004) Simultaneous detection of Entamoeba histolytica, Giardia lamblia, and Cryptosporidium parvum in fecal samples by using multiplex real-time PCR. J Clin Microbiol 42: 1220-1223.
29. Almeida A, Pozio E, Caccio SM (2010) Genotyping of Giardia duodenalis cysts by new real-time PCR assays for detection of mixed infections in human samples. Appl Environ Microbiol 76: 1895-1901.
30. Verweij JJ, Laeijendecker D, Brienen EA, van Lieshout L, Polderman AM (2003) Detection of Cyclospora cayetanensis in travellers returning from the tropics and subtropics using microscopy and real-time PCR. Int J Med Microbiol 293: 199-202.

31. ten Hove RJ, van Lieshout L, Brienen EA, Perez MA, Verweij JJ (2008) Real-time polymerase chain reaction for detection of Isospora belli in stool samples. Diagn Microbiol Infect Dis 61: 280-283.
32. Basuni M, Muhi J, Othman N, Verweij JJ, Ahmad M, et al. (2011) A pentaplex real-time polymerase chain reaction assay for detection of four species of soil-transmitted helminths. Am J Trop Med Hyg 84: 338-343.
33. Wiria AE, Prasetyani MA, Hamid F, Wammes LJ, Lell B, et al. (2010) Does treatment of intestinal helminth infections influence malaria? Background and methodology of a longitudinal study of clinical, parasitological and immunological parameters in Nangapanda, Flores, Indonesia (ImmunoSPIN Study). BMC Infect Dis 10: 77.
34. Verweij JJ, Canales M, Polman K, Ziem J, Brienen EA, et al. (2009) Molecular diagnosis of Strongyloides stercoralis in faecal samples using real-time PCR. Trans R Soc Trop Med Hyg 103: 342-346.
35. ten Hove RJ, Verweij JJ, Vereecken K, Polman K, Dieye L, et al. (2008) Multiplex real-time PCR for the detection and quantification of Schistosoma mansoni and S. haematobium infection in stool samples collected in northern Senegal. Trans R Soc Trop Med Hyg 102: 179-185.
36. Rolfe KJ, Parmar S, Mururi D, Wreghitt TG, Jalal H, et al. (2007) An internally controlled, one-step, real-time RT-PCR assay for norovirus detection and genogrouping. J Clin Virol 39: 318-321.
37. Rousselon N, Delgenes JP, Godon JJ (2004) A new real time PCR (TaqMan PCR) system for detection of the16S rDNA gene associated with fecal bacteria. J Microbiol Methods 59: 15-22.
38. Liu J, Kibiki G, Maro V, Maro A, Kumburu H, et al. (2011) Multiplex reverse transcription PCR Luminex assay for detection and quantitation of viral agents of gastroenteritis. J Clin Virol 50: 308-313.
