# Supplementary material for: Optimization of Quantitative PCR Methods for Enteropathogen Detection
Source: PLoS One. 2016 Jun 23;11(6):e0158199. doi: 10.1371/journal.pone.0158199 (PMC4918952; doi:10.1371/journal.pone.0158199)
Supplement: S2 Table — (PDF) [file pone.0158199.s002.pdf]

S2 Table. List of genomic material included in the specificity testing of all the assays.

Organism	Genomic material
Adenovirus	Adenovirus 1 (ATCC VR-1D)
	Adenovirus 5 (ATCC VR-5D)
	Adenovirus 40 (ATCC VR-931D)
	Adenovirus 41 (ATCC VR-930D)
Aeromonas spp	*Aeromonas hydrophila* (ATCC7965 and 7966)
	Aeromonas cavae (ATCC 15468)
	Aeromonas veronii (ATCC 35624)
Ancyclostoma duodenale	Amplicon
Ascaris lumbricoides	Amplicon
Astrovirus	IVT RNA
Bacteriodes fragilis	*Bacteriodes fragilis* (ATCC 25858)
	Bacteriodes fragilis (ATCC 43858)
Blastocystis spp	*Blastocystis hominis* (ATCC 50608D)
Clostridium difficile	Clinical isolate
Campylobacter spp	*Campylobacter coli* (ATCC 43473)
	Campylobacter jejuni (ATCC 33291)
	Campylobacter hyointestinalis (ATCC 35217)
	Campylobacter upsalensis (ATCC BAA-1059)
Cryptosporidium spp	*Cryptosporidium hominis* isolate TU502 (NR-2520)
	Cryptosporidium meleagridis isolate TU1867 (NR-2521)
	Cryptosporidium parvum (Waterborne Inc)
Cyclospora cayetanensis	Amplicon
Cystoisospora belli	Amplicon
Cytomegalovirus	HCMV strain AD-169 (ATCC VR-538D)
Ebola virus	Zaire Ebola virus (NR-31806)
Encephalitozoon intestinalis	*Encephalitozoon intestinalis* (Waterborne Inc)
Entamoeba spp	*Entamoeba dispar* (clinical isolate)
	Entamoeba bangladeshi (clinical isolate)
	Entamoeba histolytica HM1
	Entamoeba moshkovskii (clinical isolate)
Enterocytozoon bieneusi	Amplicon
Enterovirus	Enterovirus 71 (NR-4961)
Epstein-Barr virus	EBV infected HCC2157BL (ATCC CRL-2341D)
Escherichia coli	EAEC O42, EAEC JM221, EAEC 144-1-1, EAEC HS, EAEC495-1
	EHEC O157:H7
	EIEC O124
	EPEC O127:H6 E2348/69
	ETEC H10407
Giardia lamblia	*Giardia lamblia* assemblage A
	Giardia lamblia assemblage B
Helicobacter pylori	Helicobacter pylori (ATCC 43504)
Human herpesvirus 6	Amplicon
Human herpesvirus 7	Amplicon
Listeria monocytogenes	Listeria monocytogenes strain J0161 (NR-13342)
MS2	MS2 (ATCC 15597-B1)
Mycobacterium tuberculosis	Mycobacterium tuberculosis H37RV
Necator americanus	Amplicon
Norovirus GI	IVT RNA
Norovirus GII	IVT RNA
PhHV	PhHV
Plesiomonas shigelloides	Plesiomonas shigelloides (ATCC 51903D)
Rotarix	IVT RNA
RotaTeq	IVT RNA
Rotavirus	Rotavirus G1P[8] wa
	Rotavirus G2P[4] DS-1
Salmonella enterica	*Salmonella enterica* serovar collection (*Salmonella* Reference Collection B)
Sapovirus	IVT RNA, clinical samples
Schistosoma mansoni	*Schistosoma mansoni* strain NMRI (NR-28910)
Shigella spp	*Shigella boydii* (clinical isolate)
	Shigella dysenteriae (clinical isolate)
	Shigella flexneri (clinical isolate)
	Shigella sonnei (clinical isolate)
Strongyloides stercoralis	Amplicon
Trichuris trichuria	Amplicon
Vibrio cholerae	*Vibrio cholerae* (ATCC 9458 and 582)
Vibrio parahaemolyticus	*Vibrio parahaemolyticus* (ATCC 17802)

Yersinia spp	*Yersinia enterocolitica* (ATCC 23715)<br>*Yersinia pseudotuberculosis* (ATCC 6903)
